# Sport practice and plantar pressure in children aged 10–18 years: evaluation using Namrol® Podoprint*®*

**DOI:** 10.1080/07853890.2021.1896796

**Published:** 2021-09-28

**Authors:** C. Lopes, J. Neves Silva, E. Matos, S. Xavier Sousa, D. Gonçalves, N. Azevedo, L. Rodrigues, G. Pacheco

**Affiliations:** ISAVE – Instituto Superior de Saúde, CICS – Centro Interdisciplinar em Ciências da Saúde, Portugal

## Abstract

**Introduction:**

The foot is key to sport [1] and is part of a set of mechanisms responsible for absorbing impacts, maintaining balance and distribution of forces. Therefore, special attention to the distribution of plantar pressure is necessary [2]. Baropodometry consists of the quantification of anteroposterior and lateral oscillations of the sole of the foot, while the individual remains under a pressure platform [3]. The goal of this investigation is to verify if there is an association between students' sports practice and their distribution of plantar pressure and to analyse whether there are differences regarding plantar pressure distribution between students who practice and those who do not practice a specific modality (volleyball, swimming or soccer).

**Material and methods:**

The current research is a cross-sectional study carried out in a non- probabilistic sample comprising 499 students from Amares, Portugal aged 10–18 years (average age: 13.79 ± 2.50), 238 (47.7%) being males and 261 (52.3%) females. An informed consent was given to all participants and a questionnaire was used to ascertain whether individuals practiced physical exercise and, if so, what type of exercise they practiced. Finally, an evaluation of the plantar pressure distribution of the study participants was made using the Namrol® Podoprint® baropodometry platform. All data were analysed using descriptive and inferential statistics, which were performed using the Mann–Witney (*U*) test for association. Significance levels (denoted as *α* or alpha) of 0.05 and 0.01 have been considered for the presence of statistically significant association between the considered variables.

**Results:**

There was no statistically significant association between sport practice and the presence of pressure changes in the plantar area of the students analysed. Regarding the students who practice volleyball, we found statistically significant differences in the right/left pressure distribution, with a predominance of pressure in the right foot (*U* = 12327.000; *Z*= − 1.968; *p*-value = .049).

**Discussion and conclusion:**

This study verified that there is no statistically significant association between the practice of sport and the presence of changes in the plantar zone when comparing students who practice sports and those who do not. It was verified that the volleyball practitioners presented a predominance of plantar pressure in the right foot, in contrast with the non-practitioners, which showed an equal distribution of plantar pressure. In this sense, future studies comprising a larger sample of participants of different sports, namely volleyball, may help identify which technical movements may be contributing to these baropodometric imbalances. In addition, these studies are important to help develop strategies that counterbalance these differences in foot pressure of children, minimising the future appearance of postural changes and related problems.
